# Risk factors influencing the prognosis of elderly patients infected with COVID-19: a clinical retrospective study in Wuhan, China

**DOI:** 10.18632/aging.103631

**Published:** 2020-07-11

**Authors:** Shan Gao, Fang Jiang, Wei Jin, Yuan Shi, Leilei Yang, Yanqiong Xia, Linyan Jia, Bo Wang, Han Lin, Yin Cai, Zhengyuan Xia, Jian Peng

**Affiliations:** 1 Department of Anesthesiology, Wuhan Third Hospital, Tongren Hospital of Wuhan University, Wuhan, Hubei, China; 2 Department of Anesthesiology, The University of Hong Kong, Hong Kong, China; 3 Department of Pain Medicine, Wuhan Third Hospital, Tongren Hospital of Wuhan University, Wuhan, Hubei, China; 4 Department of Anesthesiology, Affiliated Hospital of Guangdong Medical University, Zhanjiang, China; 5 Department of Anesthesiology, The Second Affiliated Hospital and Yuying Children's Hospital of Wenzhou Medical University, Wenzhou, China

**Keywords:** COVID-19, elderly patients, risk factors, C-reactive protein, comorbidities

## Abstract

The mortality rate of elderly patients with Coronavirus Disease 2019 (COVID-19) was significantly higher than the overall mortality rate. However, besides age, leading death risk factors for the high mortality in elderly patients remain unidentified. This retrospective study included 210 elderly COVID-19 patients (aged ≥ 65 years), of whom 175 patients were discharged and 35 died. All deceased patients had at least one comorbidity. A significantly higher proportion of patients in the deceased group had cardiovascular diseases (49% vs. 20%), respiratory diseases (51% vs. 11%), chronic kidney disease (29% vs. 5%) and cerebrovascular disease (20% vs. 3%) than that in the discharged group. The median levels of C-reactive protein (125.8mg/L vs. 9.3mg/L) and blood urea nitrogen (7.2mmol/L vs. 4.4mmol/L) were significantly higher and median lymphocyte counts (0.7×10^9^/L vs. 1.1×10^9^/L) significantly lower in the deceased group than those in the discharged group. The survival curve analysis showed that higher C-reactive protein (≥5mg/L) plus any other abnormalities of lymphocyte, blood urea nitrogen or lactate dehydrogenase significantly predicted poor prognosis of COVID-19 infected elderly patients. This study revealed that the risk factors for the death in these elderly patients included comorbidities, increased levels of C-reactive protein and blood urea nitrogen, and lymphopenia during hospitalization.

## INTRODUCTION

Since the outbreak in December 2019, COVID-19 caused by the severe acute respiratory syndrome coronavirus 2 (SARS-CoV-2), has rapidly grown into a pandemic worldwide [1–3]. While the overall mortality rate during the early phase of the pandemic in both China and Italy was around 2.3% [1, 4, 5], the mortality rate was significantly higher in the elderly especially in those aged 65 years or older. A report from the United States Centers for Disease Control and Prevention COVID-19 response team showed that 80% of deaths associated with COVID-19 were among adults aged ≥65 years [6], which is similar to that initially reported from China regarding the high mortality rate in elderly patients with COVID-19 [7, 8]. Available evidence suggest that old age *per se*, especially aged ≥65 years [9], is an independent risk factor for COVID-19 related mortality irrespective of whether there may exist underlying comorbidities. In addition, the majority of the persons aged ≥65 years may have one or more pre-existing complications which would further increase the rate of mortality. At present, clinical research on COVID-19 has been mainly focused on the epidemiological characteristics, clinical manifestations, prognosis of the general population or comparisons between aged or young populations [9]. However, studies that specifically aimed to identify fatal risk factors for elderly COVID-19 patients (aged ≥65 years) are rare. Of note, a recent study showed that despite both age≥ 65 years and pre-existing comorbidities, including hypertension or diabetes, were independently associated with the development of acute respiratory distress syndrome (ARDS), age ≥ 65 years was a major risk factor associated with the progression from ARDS to death [9]. However, the main risk factors that are responsible for the death of elderly infected with SARS-CoV-2 have yet to be determined. Here, we report on the characteristics of the largest cohort of elderly COVID-19 patients in Wuhan city, China, the epicenter of the SARS-CoV-2 outbreak, and describe the fatal risk factors for the most fragile elderly patients with COVID-19 infection in the hope that this will help to guide the clinicians to identify the elderly people who are at higher danger to progress to severe illness from COVID-19 infection at an early stage and adjust treatment plans to reduce mortality.

## RESULTS

### Demographics and characteristics

A total of 302 patients aged ≥65 years old admitted between January 23, 2020 to February 29, 2020 were screened, and excluded 69 suspected cases admitted without laboratory confirmation tests for SARS-CoV-2, as were 9 cases transferred to Huoshenshan Hospital or Leishenshan Hospital, and 14 patients with only one laboratory test during hospitalization. Overall, 210 patients were finally included in this study (Table 1). The median age of the 210 patients was 71 (interquartile range [IQR] 67-77) years, and the ratio of male and female was approximate equal. 175 patients were in the discharged group, with a median age was 70 (IQR 67-74) years and 79 (45%) were male, while 35 patients were in the deceased group, with a median age was 74 (IQR 70-82) years, 22 (63%) were male. Patients in the deceased group were significantly older (74 years, IQR 70-82) than that in the discharged group (70 years, IQR 67-74). In the deceased group, the median time from onset of symptoms to admission and death were, respectively, 8 (IQR 6-14) days and 14 (IQR 12-24) days. In the discharged group, the median time from onset of symptoms to admission and discharge were 10 (IQR7-15) days and 26 (IQR21-29) days, respectively. A total of 18 cases required intensive care admission, with 16 cases in the deceased group.

**Table 1:** Baseline characteristics, treatments, complications of patients infected with COVID-1.

	**Total (n=210)**	**Discharged group (n=175)**	**Deceased group (n=35)**	**P Value^a^**
Age, median (IQR), y	71 (67-77)	70 (67-74)	74 (70-82)	<0.001
Sex, n (%)				
Male	101 (48)	79 (45)	22 (63)	0.056
Female	109 (52)	96 (55)	13 (37)	
Duration from onset of symptoms to admission, median (IQR), d	10 (7-15)	10 (7-15)	8 (6-14)	0.889
Duration from admission to outcome, median (IQR), d	14 (10-17)	14 (11-17)	9 (5-15)	<0.001
Duration from onset of symptoms to outcome, median (IQR), d	23 (17-28)	26 (21-29)	14 (12-24)	<0.001
ICU cases, n (%)	18/198 (9)	2/167 (1)	16/31 (52)	<0.001
Comorbidities, n (%)	159 (76)	124 (71)	35 (100)	<0.001
Hypertension	115 (55)	97 (55)	18 (51)	0.664
Diabetes	38 (18)	29 (17)	9 (26)	0.200
Cardiovascular disease	52 (25)	35 (20)	17 (49)	<0.001
COPD	3 (1)	2 (1)	1 (3)	0.435
Respiratory disease	38 (18)	20 (11)	18 (51)	<0.001
Cerebrovascular disease	13 (6)	6 (3)	7 (20)	<0.001
Chronic liver disease	18 (9)	12 (7)	6 (17)	0.047
Digestive diseases	21 (10)	15 (9)	6 (17)	0.123
Chronic kidney disease	18 (9)	8 (5)	10 (29)	<0.001
Malignancy	6 (3)	3 (2)	3 (9)	0.026
Number of Comorbidities, n (%)				
None	51 (24)	51 (29)	0 (0)	<0.001
One	56 (27)	48 (27)	8 (23)	
Two	60 (29)	54 (31)	6 (17)	
Three	22 (10)	16 (9)	6 (17)	
Four	13 (6)	4 (2)	9 (26)	
Five	4 (2)	1 (1)	3 (9)	
Six or more	4 (2)	1 (1)	3 (9)	
Signs and symptoms, n (%)				
Fever	151 (72)	122 (70)	29 (83)	0.115
Cough	148 (71)	118 (67)	30 (87)	0.031
Headache	13 (6)	10 (6)	3 (9)	0.523
Pharyngalgia	20 (10)	18 (10)	2 (6)	0.400
Fatigue	73 (35)	64 (37)	9 (26)	0.219
Anorexia	23 (11)	19 (11)	4 (11)	0.921
Nausea or vomiting	11 (5)	9 (5)	2 (6)	0.890
Myalgia	12 (6)	11 (6)	1 (3)	0.426
Chest stuffiness	76(36)	65(37)	11 (31)	0.522
Dyspnea	17 (8)	12 (7)	5 (14)	0.142
Diarrhea	24 (11)	21 (12)	3 (9)	0.562
First symptom, n (%)				
Fever	107 (51)	88 (50)	19 (54)	<0.001
Cough	65 (31)	55 (31)	10 (28)	
Pharyngalgia	16 (8)	14 (8)	2 (6)	
Fatigue	9 (4)	9 (5)	0 (0)	
Anorexia	1 (0.5)	1 (0.5)	0 (0)	
Chest tightness	6 (3)	4 (3)	2 (6)	
Dyspnea	1 (0.5)	1 (0.5)	0 (0)	
Diarrhea	5 (2)	3 (2)	2 (6)	
Temperature, °C	36.8 (36.5-37.0)	36.7 (36.5-36.9)	37.0 (36.5-37.8)	0.069
Heart rate, median (IQR), beat per minute	80 (78-88)	80 (78-86)	85 (80-104)	0.016
Respiratory rate, median (IQR), beat per minute	20 (20-22)	20 (20-22)	22 (20-26)	0.008
Mean arterial pressure, median (IQR), mm Hg	97 (92-105)	97 (93-104)	100 (92-109)	0.585
Treatment, n (%)				
Antiviral therapy	179/198 (90)	148/167 *(89)	31/31* (100)	0.048
Antibiotic therapy	163/198 (82)	133/167 (80)	30/31 (97)	0.022
Glucocorticoid therapy	65/198 (33)	42/167 (25)	23/31 (74)	<0.001
Gamma globulin therapy	37/198 (19)	23/167 (14)	14/31 (45)	<0.001
Albumin therapy	22/198 (11)	10/167 (6)	12/31 (39)	<0.001
Oxygen inhalation	133/198 (67)	103/167 (62)	30/31 (97)	<0.001
Mechanical ventilation	21/198 (11)	1/167 (1)	20/31 (66)	<0.001
CRRT	3/198 (2)	1/167 (1)	2/31 (7)	0.014
Complications, n (%)				
ARDS	27 (13)	2 (1)	25 (71)	<0.001
Acute renal failure	4 (2)	0 (0)	4 (11)	<0.001
Cerebral infarction	3 (1)	0 (0)	3 (9)	<0.001

Abbreviations: COVID-19, coronavirus disease 2019; IQR, interquartile range; ICU, intensive care unit; COPD, chronic obstructive pulmonary diseases; CRRT, continuous renal replacement therapy; ARDS, acute respiratory distress syndrome. ^a^ P values indicate differences between the discharged group and the deceased group. P < 0.05 was considered statistically significant. *Record/data were missing in 8 patients in the discharged group, and in 4 patients in the deceased group regarding whether or not the patients received a treatment.

Among the elderly patients, 159 (76%) had comorbidities, with hypertension (115 [55%]) and cardiovascular disease (52 [25%]) being the most common comorbidities, followed by diabetes (38 [18%]), respiratory disease (38 [18%]), and digestive disease (21 [10%]). There were 124 (71%) cases having comorbidities in the discharged group compared with 35 (100%) cases in the deceased group. Additionally, significantly higher percentage of patients in the deceased group had cardiovascular disease (17 [49%] vs. 35 [20%]), respiratory disease (18 [51%] vs. 20 [11%]), cerebrovascular disease (7 [20%] vs. 6 [3%]), chronic liver disease (6 [17%] vs. 12 [7%]), chronic kidney disease (10 [29%] vs. 8 [5%]) or malignancy (3 [9%] vs. 3 [2%]) than in the discharged group (Table 1).

Of the hospitalized elderly patients, the most common symptoms were fever (72%) and cough (71%), followed by chest stuffiness (36%), fatigue (35%), anorexia (11%), diarrhea (11%), pharyngalgia (10%), dyspnea (8%), headache (6%), myalgia (6%), and nausea or vomiting (5%). Among all the patients, half of them had fever (51%) as the first symptom, nearly one third had cough (31%), and a small proportion had pharyngalgia (8%), fatigue (4%), chest tightness (3%), diarrhea (2%), anorexia (0.5%), dyspnea (0.5%) as the first symptom.

### Treatment and complications

Most of the elderly patients received antiviral therapy (90%), antibiotic therapy (82%), oxygen inhalation (67%), and one third of patients were treated with glucocorticoid (33%), part of the patients received gamma globulin therapy (19%), albumin therapy (11%), mechanical ventilation (11%) or continuous renal replacement therapy (CRRT) treatment (2%). Of all the patients, ARDS (13%, 27 of 210) was the most frequently complication, followed by acute renal failure (2%) and large cerebral infarction (1%) (Table 1). 71% (25/35) of patients in the deceased group developed ARDS as compared to 2 (1%) in the discharged group. And typical pulmonary Computed Tomographic (CT) changes from a deceased and a discharged patient were shown in Supplementary Figure 1A–1F.

### Laboratory findings

Comparison of laboratory findings within 24 hours at admission were shown in Table 2. The median leucocyte counts (6.4×10^9^/L) in patients in the deceased group were higher than those in the discharged group (5.1×10^9^/L). Among them, 9 (26%) patients had leucocytes counts above the normal range in the deceased group, compared with 3 (2%) in the discharged group. The concentrations of C-reactive protein in the deceased group were significantly higher than that in the discharged group, and the levels of C-reactive protein in deceased patients were all elevated beyond the normal range. The lymphocyte counts of the deceased group were progressively decreased compared with that of the discharged group, and 60% patients in the deceased group had lymphopenia while 18% patients in the discharged group had lymphopenia. The neutrophil counts in the deceased group were higher than that in the discharged group, and 43% cases in the deceased group had neutrophil counts above the normal range as compared to 6% in the discharged group. Compared with discharged group, the platelet counts were significantly lower in the deceased group.

**Table 2:** Laboratory findings of patients infected with COVID-19.

**Laboratory finding within 24 hours on admission**	**normal range**	**Median (IQR)**			**P value^a^**
		**Total (n=210)**	**Discharged group (n=175)**	**Deceased group (n=35)**	
Leucocytes, ×10^9^/L, n (%)	(3.5-9.5) ×10^9^/L	5.2 (3.9-6.4)	5.1 (3.9-6.1)	6.4 (4.1-10.7)	0.037
<3.5 ×10^9^/L		30 (14)	24 (14)	6 (17)	<0.001
3.5-9.5 ×10^9^/L		168 (80)	148 (84)	20 (57)	
≥9.5		12 (6)	3 (2)	9 (26)	
C-reactive protein, mg/L, n (%)	(0-5) mg/L	15.5 (3.2-63.6)	9.3 (2.6-37.2)	125.8 (49.1-200.0)	<0.001
0-5 mg/L		68 (32)	68 (39)	0 (0)	<0.001
≥5 mg/L		142 (68)	107 (61)	35 (100)	
Lymphocyte, ×10^9^/L, n (%)	(1.1-3.2) ×10^9^/L	1.1 (0.8-1.5)	1.1 (0.9-1.6)	0.7 (0.4-0.9)	<0.001
<0.8 ×109/L		52 (25)	31 (18)	21 (60)	<0.001
0.8-1.1 ×109/L		42 (20)	35 (20)	7 (20)	
1.1-3.2×109/L		114 (54)	107 (61)	7 (20)	
≥3.2×109/L		2 (1)	2 (1)	0 (0)	
Neutrophils, ×10^9^/L, n (%)	(1.8-6.3) ×10^9^/L	3.3 (2.4-4.5)	3.0 (2.4-4.3)	5.2 (2.7-10.0)	0.003
<1.8 ×109/L		20 (10)	16 (9)	4 (11)	<0.001
1.8-6.3 ×109/L		165 (78)	149 (85)	16 (46)	
≥6.3 ×109/L		25 (12)	10 (6)	15 (43)	
NLR		2.9 (1.9-5.1)	2.8 (1.8-3.8)	8.4 (3.1-13.1)	<0.001
Monocytes, ×10^9^/L	(0.1-0.6) ×10^9^/L	0.4 (0.3-0.5)	0.4 (0.3-0.5)	0.4 (0.2-0.5)	0.225
Procalcitonin, ng/mL, n (%)	(0.02-0.05) ng/mL				
<0.02 ng/mL		0	0	0	<0.001
0.02-0.05 ng/mL		140/205 (68)	135/170 (79)	5 (14)	
≥0.05 ng/mL		65/205 (32)	35/170 (21)	30 (86)	
Platelet count, ×10^9^/L, n (%)	(125-350) ×10^9^/L	206.0 (159.8-267.0)	216.0 (169.0-285.0)	168.0 (124.0-216.0)	<0.001
<125 ×109/L		20/208 (10)	19/174 (11)	1/34 (3)	0.010
125-350 ×109/L		164/208 (79)	139/174 (80)	25/34 (74)	
≥350 ×109/L		24/208 (11)	16/174 (9)	8/34 (23)	
ALT, IU/L, n (%)	(7-40) IU/L	29.5 (17.0-45.5)	27.0 (17.0-43.0)	35.0 (18.0-77.0)	0.427
<7 IU/L		1/208 (0.5)	0/173 (0)	1 (3)	0.261
7-40 IU/L		140/208 (67)	121/173 (70)	19 (54)	
≥40 IU/L		67/208 (32.5)	52/173 (30)	15 (43)	
AST, IU/L, n (%)	(0-45) IU/L	26.0 (21.0-38.3)	25.0 (20.0-35.5)	45.0 (26.0-72.0)	0.008
<45 IU/L		165/208 (79)	149/173 (86)	16 (46)	<0.001
≥45 IU/L		43/208 (21)	24/173 (14)	19 (54)	
Cr, μmol/L, n (%)	(40-105) μmol/L	67.1 (56.5-84.1)	65.2 (55.2-80.2)	76.0 (64.0-107.4)	0.012
<40 μmol/L		2/208 (1)	1/173 (0.5)	1 (3)	0.139
40-105μmol/L		197/208 (95)	167/173 (96.5)	30 (86)	
≥105μmol/L		9/208 (4)	5/173 (3)	4 (11)	
BUN, mmol/L, n (%)	(3.1-7.2) mmol/L	4.7 (3.4-6.1)	4.4 (3.3-5.6)	7.2 (5.0-11.1)	<0.001
<3.1 mmol/L		28 (13)	26 (15)	2 (5)	<0.001
3.1-7.2 mmol/L		146 (70)	130 (74)	16 (46)	
≥7.2 mmol/L		36 (17)	19 (11)	17 (49)	
CK, IU/L, n (%)	(30-180) IU/L	65.0 (43.0-116.0)	60.0 (41.0-91.5)	137.0 (54.0-363.0)	0.008
<30 IU/L		19/208 (9)	16/173 (9)	3 (9)	<0.001
30-180 IU/L		162/208 (78)	144/173 (83)	18 (51)	
≥180 IU/L		27/208 (13)	13/173 (8)	14 (40)	
CK-MB, IU/L, n (%)	(0-25) IU/L	10.0 (7.0-13.0)	9.0 (7.0-12.0)	13.0 (8.0-19.0)	0.002
<25 IU/L		204/208 (98)	171/173 (99)	33 (94)	0.074
≥25 IU/L		4/208 (2)	2/173 (1)	2 (6)	
APTT, s	(21-35) s	27.6 (23.7-31.4)	27.3 (23.5-30.6)	31.3 (26.0-35.4)	0.012
Prothrombin time, s	(10-13) s	11.6 (11.0-12.4)	11.6 (10.9-12.3)	12.1 (11.5-12.8)	0.035
Thrombin time, s	(13-21) s	19.5 (16.4-22.2)	19.6 (14.9-22.3)	18.7 (17.4-21.4)	0.180
D-dimer, μg/mL, n (%)	(<0.5) μg/mL	0.6 (0.2-1.9)	0.6 (0.2-1.1)	3.6 (0.6-5.7)	<0.001
0-0.5 μg/mL		42/168 (25)	40/139 (29)	2/29 (7)	0.014
≥0.5 μg/mL		126/168 (75)	99/139 (71)	27/29 (93)	
Albumin, g/L, n (%)	(40-55) g/L	35.7 (31.9-39.3)	36.2 (32.8-39.6)	31.2 (26.7-34.6)	<0.001
<30 g/L		30/206 (15)	17/172 (10)	13/34 (38)	<0.001
Glucose, mmol/L, n (%)	(3.9-6.1) mmol/L	5.7 (4.7-7.4)	5.5 (4.7-6.9)	6.9 (5.0-7.9)	0.105
<3.9 mmol/L		2/205 (1)	2/171 (1)	0/34 (0)	<0.001
3.9-6.1 mmol/L		123/205 (60)	112/171 (66)	11/34 (32)	
≥6.1 mmol/L		80/205 (39)	57/171 (33)	23/34 (68)	
Total bilirubin, μmol/L	(2-21) μmol/L	8.9 (6.6-11.9)	8.4 (6.5-11.6)	9.6 (6.7-16.7)	0.043
Triglyceride, mmol/L	(0.5-1.72) mmol/L	1.2 (0.9-1.7)	1.2 (0.9-1.6)	1.3 (0.9-1.9)	0.635
Total cholesterol, mmol/L	(3.1-5.7) mmol/L	3.9 (3.3-4.6)	4.0 (3.5-4.6)	3.4 (2.8-4.2)	0.051
Lactate dehydrogenase, IU/L, n (%)	(114-240) IU/L	208.0 (165.8-270.8)	199.0 (164.0-244.0)	367.0 (251.0-547.0)	<0.001
<240 IU/L		128/205 (62)	121/171 (71)	7/34 (21)	<0.001
≥240 IU/L		77/205 (38)	50/171 (29)	27/34 (79)	

Abbreviations: COVID-19, coronavirus disease 2019; IQR, interquartile range; NLR, neutrophil-to-lymphocyte ratio; ALT, alanine aminotransferase; AST, aspartate aminotransferase; Cr, serum creatinine; BUN, blood urea nitrogen; CK, creatine kinase; CK-MB, creatine kinase isoenzyme; APTT, activated partial thromboplastin time. ^a^ P values indicate differences between the discharged group and the deceased group. P < 0.05 was considered statistically significant.

Biochemical test results were shown in Table 2. The concentrations of aspartate aminotransferase (AST), serum creatinine (Cr), and blood urea nitrogen (BUN) of the deceased group were all significantly higher than that of the discharged group. And, nearly half (49%) of the deceased patients had BUN concentrations elevated beyond the normal range as compared to 11% of the patients in the discharged group. The levels of creatine kinase (CK) and creatine kinase isoenzyme (CK-MB) in the deceased group were significantly higher than those in the discharged group. Significantly more patients in the deceased group (86%) had procalcitonin concentrations above the normal range than in discharged group (21%, 35 of 170).

The concentrations of lactate dehydrogenase (LDH) were significantly higher in the deceased group than those in the discharged group, with 79% (27 of 34) deceased patients had LDH concentrations above the normal range as compared to 29% (50 of 171) in the discharged group. The median concentrations of fasting blood glucose did not differ significantly between the two groups, however relatively more patients in the deceased group (68%, 23 of 34) had acute hyperglycemia (glucose ≥6.1 mmol/L) as compared to 33% (57 of 171) in the discharged group. Albumin concentrations were significantly lower in the deceased group than in the discharged group, with 38% (13 of 34) deceased patients and 10% (17 of 172) discharged patients developed hypoalbuminemia. In addition, D-dimer level in the deceased patients was significantly higher than that in the discharged patients. The median activated partial thromboplastin time and prothrombin time as well as the total bilirubin concentrations in the deceased group were all significantly higher than those in the discharged group. Monocytes count, concentrations of alanine aminotransferase (ALT), triglyceride and thrombin time did not significantly differ between the two groups.

### Receiver operating characteristic curve, survival curve and dynamic profile

**Figure 1:**
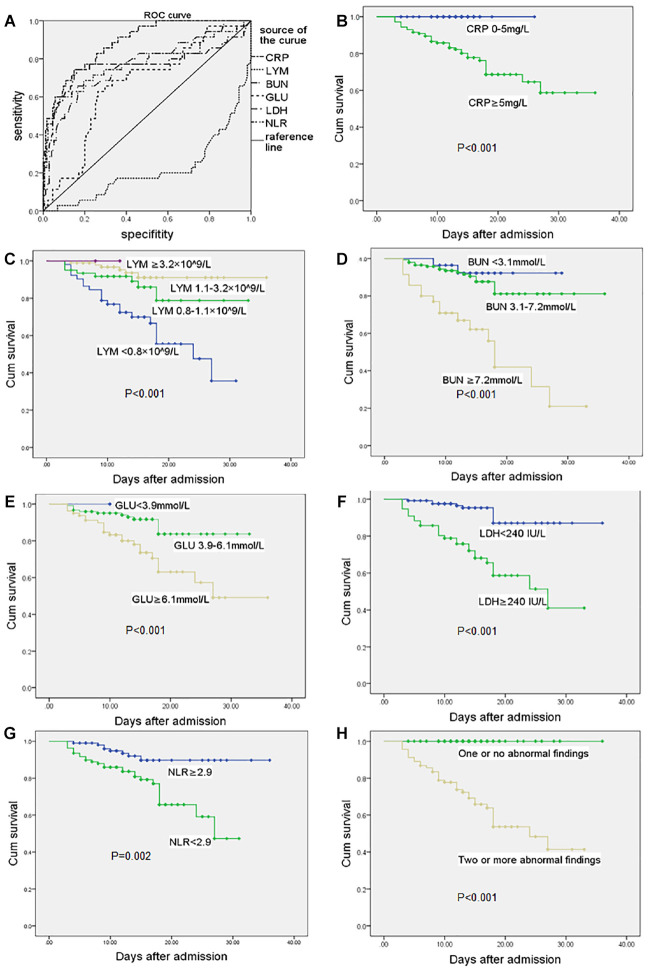
**Receiver operating characteristic curve and survival curve.** (**A**) ROC in CRP, LYM, BUN, GLU, LDH, NLR at admission. Survival curves in elderly COVID-19 patients with different levels of CRP (**B**), LYM(**C**), BUN (**D**), GLU (**E**), LDH (**F**), NLR (**G**, NLR value take median value in total patients) at admission. (**H**) Two or more abnormal values of CRP, LYM, BUN, LDH in the patients at admission can significantly predict poor prognosis of COVID-19 infected elderly patients. Abbreviations: COVID-19, coronavirus disease 2019; ROC, receiver operating curve; CRP, C-reactive protein; LYM, lymphocytes; BUN, blood urea nitrogen; GLU, glucose; LDH, lactate dehydrogenase; NLR, neutrophil-to-lymphocyte ratio. P-value reported in each subplot indicates the difference between survival curves by Kaplan-Meier method with log-rank test. P < 0.05 was considered statistically significant.

The relationships between routine blood test results, including blood biochemistry, inflammatory markers and the prognosis were analyzed. As shown in Figure 1A, the values of area under curve (AUC) of C-reactive protein, lymphocytes, BUN, glucose, LDH, and neutrophil to lymphocyte ratio (NLR) were respectively 0.857, 0.214, 0.769, 0.660, 0.766, and 0.774. The optimal cut-off values of C-reactive protein, BUN, glucose, LDH and NLR were 63 mg/L, 6.1 mmol/L, 6.5 mmol/L, 265 IU/L, 6.48 respectively (Table 3). It showed that higher C-reactive protein, BUN, LDH and NLR on admission could significantly predict poor prognosis of COVID-19 infected elderly patients.

**Table 3:** Areas under the curve (AUC) of CRP, LYM, BUN, GLU, LDH, and NLR.

**Test result variable (s)**	**AUC**	**highest specificity**	**highest sensitivity**	**optimal cut-off values**
CRP	0.857	0.85	0.74	63 mg/L
LYM	0.214			
BUN	0.769	0.82	0.66	6.1 mmol/L
GLU	0.660	0.76	0.63	6.5 mmol/L
LDH	0.766	0.79	0.74	265 IU/L
NLR	0.774	0.69	0.60	6.48

Abbreviations: AUC, areas under the curve; BUN, blood urea nitrogen; CRP, C-reactive protein; GLU, glucose; LYM, lymphocytes; NLR, neutrophil-to-lymphocyte ratio.

Survival curves derived from C-reactive protein, lymphocyte, BUN, glucose, LDH and NLR individually and from the frequency of abnormal findings in relation to C-reactive protein, lymphocyte, BUN, and LDH were shown in Figure 1B–1H. The survival rate was much higher in patients with normal values of C-reactive protein, LDH and NLR. Abnormally high levels of BUN and glucose were associated with lower survival rate. Patients suffered severe lymphopenia had decreased survival rate, and the lower the lymphocyte count the lower the survival rate. All the deceased patients had abnormally high C-reactive protein level plus at least one abnormal value of either lymphocyte, BUN or LDH at admission. And, all elderly patients that concomitantly had abnormally high C-reactive protein plus two abnormalities of lymphocyte, BUN or LDH were in the deceased group.

**Figure 2:**
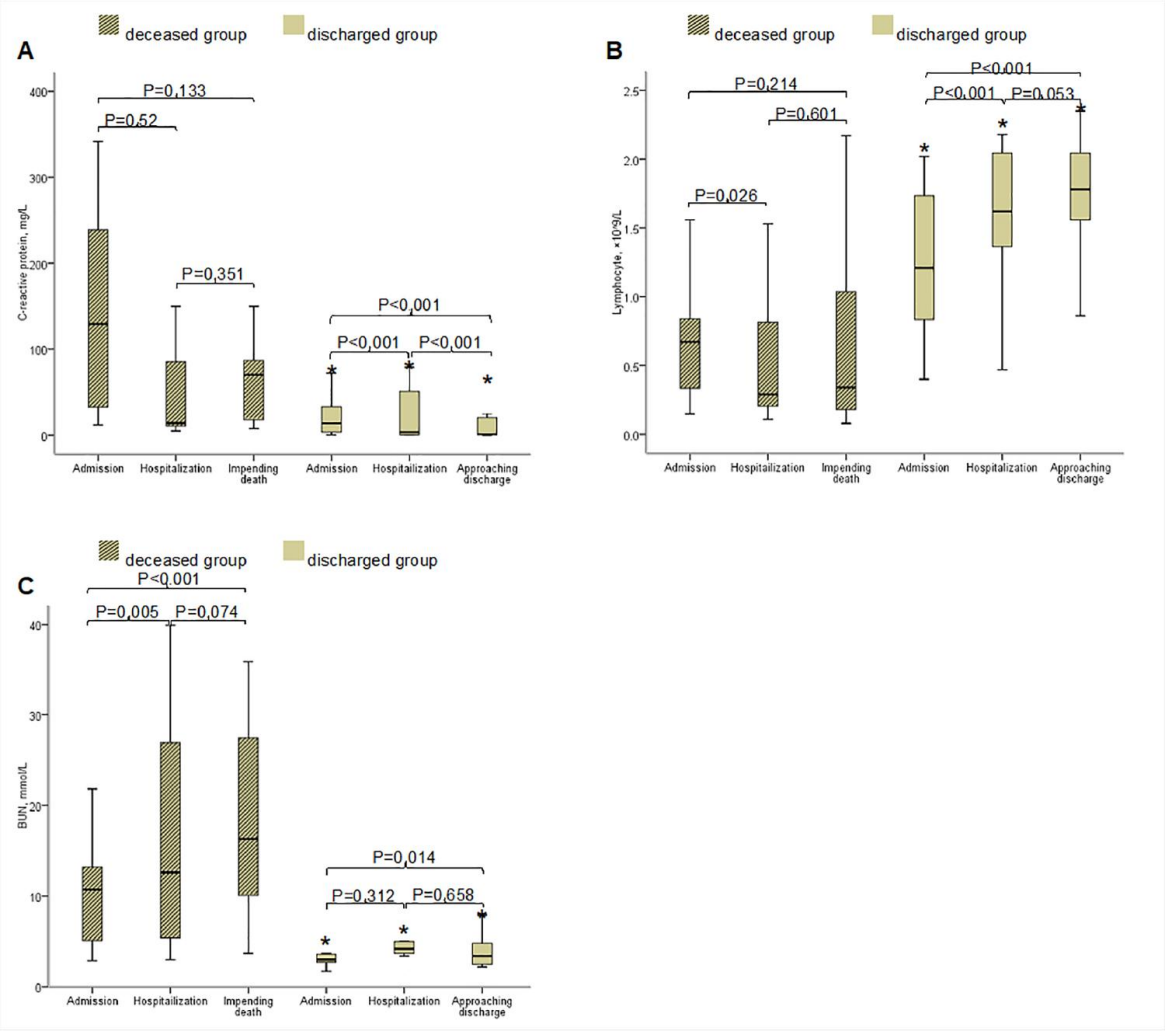
****Dynamic Changes of C-reactive protein (**A**), lymphocyte (**B**) and BUN (**C**) within 24 hours at admission, during hospitalization and before discharge or death. Abbreviations: BUN, blood urea nitrogen. The horizontal lines represent the median value in each group. P values indicate differences among admission, hospitalization, impending death between the discharged group and the deceased group. *P<0.05 vs. deceased group. P<0.05 was considered statistically significant.

Dynamic profile of the three major findings/predictors (i.e. C-reactive protein, lymphopenia and BUN), were tracked from 24 hours at admission, during hospitalization and from the last laboratory findings before discharge or death, respectively. As shown in Figure 2, at admission, all deceased patients had markedly high level of C-reactive protein than that in the discharged patients. The level of C-reactive protein slightly reduced during the time impending death, however, it was still higher than that in the discharged patients. Most deceased patients had lymphopenia at admission, and lymphopenia became more serious when approaching death. By contrast, few discharged patients had lymphopenia, and it returned to normal during hospitalization. At admission, most deceased patients had BUN above normal range as compared to that in the discharged patients, and the BUN levels increased when impending death (Figure 2).

## DISCUSSION

This study, to our knowledge, is the largest cohort study to date of elderly COVID-19 patients with definitive outcomes of the disease and describes the fatal risk factors for the most fragile elderly patients. In keeping with the findings that aging is a risk factor for patients with COVID-19 in the overall population, the median age of patients in the deceased group was significantly older than the discharged group, suggesting that the older the patients, the higher the mortality. Consistently, epidemiological studies conducted among 72,314 patients across the China showed that the mortality of patients aged 70-79 years was 8.0%, and the mortality of patients aged over 80 years was 14.8% [7]. The relatively high mortality of elderly patients in our study (16.7%, 35/210) was possibly related to the lack of medical resources caused by the outbreak of COVID-19 initially in Hubei Province, China, and similarly in Europe and north America later on. But, most likely, the high mortality in the elderly may be attributable to the lack of adequate information or experience regarding what are the most fatal risk factors for the elderly patients, in addition to the generally known risk factors such as comorbidities. The higher mortality in the elderly patients could be in part due to the hypoimmunity, as less robust immune responses in elderly patients may render them more susceptible to ARDS after SARS-CoV-2 infection and die from respiratory failure [10]. Indeed, in the present study, significantly more patients in the deceased group suffered from lymphopenia and failed to survive ICU and wean from mechanical ventilation, while those who survived usually had relatively normal lymphocyte level or lymphocyte levels could gradually recover.

A study showed that male patients accounted for 67% of critically ill patients in the general population [11]. However, our study did not identify significant gender difference between the deceased and discharged elderly. This is possibly because that female patients in our study are at postmenopausal age. SARS-CoV-2 uses angiotensin-converting enzyme 2 (ACE2) as a functional receptor [12, 13] and infects type 2 alveolar epithelial cells, which subsequently generates strong immune response and even induces cytokine storm [14]. In our study, the lymphocytes in the deceased group decreased progressively while neutrophils increased, leading to most significantly increased NLR, which is predictive of mortality. Another manifestation of cytokine storm is the elevation of C-reactive protein. In our study, the lever of C-reactive protein in the deceased group was significantly higher when compared with the discharged group. And, the receiver operating characteristic curve analysis indicates that high level of C-reactive protein is a risk factor of mortality in elderly patients. The fact that the levels of C-reactive protein significantly decreased after treatment in the discharged group but not in the deceased group (Figure 2A) provides support that the dynamic changes of C-reactive protein may serve as good indicator of prognosis of the elderly patients with COVID-19. Also, recent study showed that SARS-CoV-2 may directly affect kidney cells [15] and the myocardium [16], these may explain why high BUN and LDH are also highly predictive of mortality in the elderly, despite that LDH is a non-specific myocardial injury marker.

### Limitations

This study has several limitations. Firstly, it is a single-center, retrospective study, and included participants were elderly patients who aged over 65 years, therefore, it is limited in sample size. Secondly, elderly patients are special, especially patients with older age, may cause recall bias when conducting epidemiological investigations, especially if there are comorbidities that are used as an analysis of prognosis-related factors.

## CONCLUSIONS

This study shows that elderly patients with comorbidities had a greater risk of death, and, the enhanced level of C-reactive protein, blood urea nitrogen or lactate dehydrogenase at admission, progressively lowered lymphocyte counts during hospitalization, alone and especially in combination predict the poor prognosis in elderly patients with COVID-19.

## MATERIALS AND METHODS

### Study population

This study was a single-center, retrospective, observational study. We included elderly patients aged ≥65 years who were admitted to Wuhan Third Hospital, Wuhan, China, one of the designated hospitals for the treatment of COVID-19 assigned by the government, during the period from January 23, 2020 to February 29, 2020. For all patients, the ethics committee of Wuhan Third Hospital approved this study (Wu San Yi Lun KY2020-019) and granted a waiver of informed consent from study participants.

We included patients who were confirmed with COVID-19 according to World Health Organization interim guidance [17], and laboratory confirmation of SAR-CoV-2 was done by quantitative RT-PCR on samples from the respiratory tract, which was performed by the local health authority. Discharge criteria for patients include: body temperature returned to normal for more than 3 days; the respiratory symptoms had improved significantly, the pulmonary imaging showed a significant improvement of acute exudative lesions, and the nucleic acid test result of respiratory specimens of sputum and/or nasopharyngeal swabs became negative for two successive times (sampling interval more than 24 hours). Patients who were transferred to Huoshenshan Hospital and Leishenshan Hospital during the disease progress and thus the records were not complete at the Wuhan Third Hospital, and patients who were only subjected to one laboratory test during their admission were excluded. The included patients were divided into the discharged group and the deceased group according to the prognosis of patients.

### Data collection

A trained team of physicians and medical staffs reviewed and collected epidemiological, demographic, clinical, and prognosis data from electronic medical records, and the records were double checked and confirmed by two researchers (SG and WJ) respectively. The recorded comorbidities included hypertension, diabetes, cardiovascular diseases, chronic obstructive pulmonary diseases (COPD), respiratory diseases, cerebrovascular diseases, chronic liver disease, digestive diseases, chronic kidney disease and malignancy. The signs and symptoms including fever, cough, headache, fatigue, nausea or vomiting, anorexia, myalgia, chest stuffiness, dyspnea, and diarrhea were recorded. The patients’ life vital signs including heart rate, respiratory rate, and mean arterial pressure (MAP) were also collected.

The laboratory findings were collected within 24 hours on admission, which included leucocytes, C-reactive protein, lymphocytes, neutrophils, NLT, ALT, AST, Cr, BUN, CK, CK-MB, coagulation function, fasting blood glucose, albumin, total bilirubin, triglycerides, total cholesterol, and LDH. Lymphopenia was diagnosed as the counts of lymphocytes below 0.8 ×10^9^/L according to the Common Terminology Criteria for Adverse Events version 5.0 [18]. Hyperglycemia was defined as concentrations of fasting blood glucose above 6.1 mmol/L. Hypoalbuminemia was diagnosed as concentrations of albumin below 30 g/L according to American Society of Chest Physicians/Society of Critical Care Medicine criteria [19].

The treatments included antiviral treatment, antibiotic therapy, glucocorticoid therapy, gamma globulin therapy, albumin therapy, oxygen inhalation, mechanical ventilation, and CRRT. The duration of antiviral therapy was 7-10 days, which included the applications of oseltamivir, ganciclovir, and arbidol. While the antibiotic therapy lasted for 14 days, which included the use of cefoperazone sulbactam and moxifloxacin. No patients received treatments for specific interleukin 6 (IL-6) inhibition or anti-cytokine-storm medications. The complications included ARDS, acute renal failure, and cerebral infarction.

### Definitions

The COVID-19 onset time was defined as the date when the first sign or symptom was noticed. Acute cardiac injury was identified if the cardiac biomarkers (e.g. hypersensitive troponin I, Creatine kinase–MB) were above the 99% upper reference limit or new abnormalities were shown in electrocardiography and echocardiography [20]. Respiratory failure was identified according to the guidance of World Health Organization for COVID-19 [17]. Acute kidney injury was defined according to the KDIGO clinical practice guidelines [21]. Cerebral infarction was diagnosed according to the 2018 Stroke Guidelines [22].

### Outcomes

The primary outcomes were death and successful discharge of the patients. The second outcomes were laboratory results, radiological data, treatments, and complications of the groups and the analysis of their prognostic values.

### Statistical analysis

The categorical variables were compared by chi-square test or Fisher’s test, and expressed as frequency and percentage; the continuous variables were compared by rank sum test, and presented as median (IQR) between the discharged group and deceased group. AUC of receiver operating characteristic (ROC) was calculated to predict the prognosis of elderly patients. Survival curve was developed using the Kaplan-Meier method with log-rank test to predict death or discharge in the elderly. A two-sided P value less than 0.05 was considered statistically significant. The SPSS 21.0 software was used for all the analyses.

## Supplementary Material

Supplementary Figure 1
