# Supplementary material for: Risk factors influencing the prognosis of elderly patients infected with COVID-19: a clinical retrospective study in Wuhan, China
Source: Aging (Albany NY). 2020 Jul 11;12(13):12504–16. doi: 10.18632/aging.103631 (PMC7377843; doi:10.18632/aging.103631)
Supplement: Supplementary Figure 1 [file aging-12-103631-s001..pdf]

SUPPLEMENTARY FIGURE

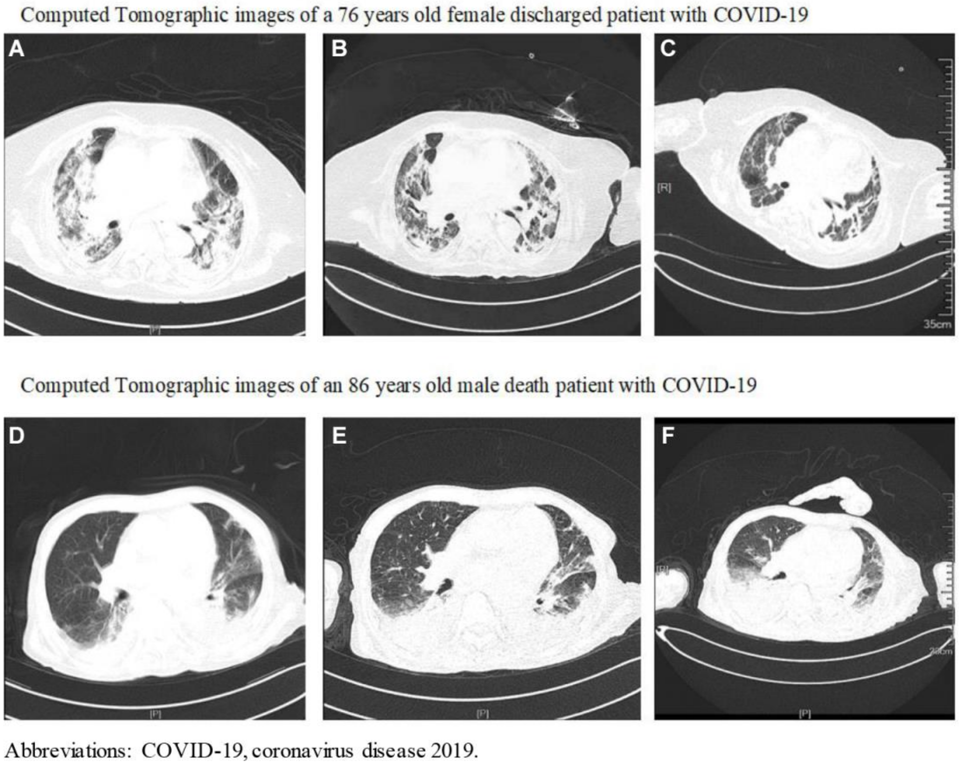

Supplementary Figure 1. Computed Tomographic (CT) findings of two patients. As shown in (**A**–**C**) a 76 years old female discharged patient, she had fever and headache for 5 days before admission on January 28, 2020. (**A**) image obtained on day 23 after symptom onset shows progressive multiple ground glass opacities, massive high-density shadows in bilateral lungs. (**B**) image obtained on day 28 after symptom onset shows multiple ground glass opacities and high-density shadows in bilateral lungs. (**C**) image obtained on day 34 after symptom onset showed that the consolidation was obviously resolved and pulmonary interstitial fibrosis attenuated. The patient was discharged on March 17, 2020, the duration from admission to discharge was 49 days, and the duration from onset of symptoms to discharge was 54 days. (**D**–**F**) an 86 years old male death patient, he had cough and chest tightness for 7 days before admission on February 25, 2020. (**D**) image obtained on day 8 after symptom onset showed multiple ground glass opacities, high-density shadows in bilateral lungs. (**E**) image obtained on day 14 after symptom onset showed progressive multiple ground glass opacities and mass shadows of high-density shadows in bilateral lungs. (**F**) image obtained on day 21 after symptom showed progressive multiple ground glass opacities and mass shadows of high-density shadows in bilateral lungs. The patient died on March 10, 2020, and the duration from admission to death was 14 days, while the duration from onset of symptoms to death was 21 days.
